# Angiogenesis in Ischemic Stroke and Angiogenic Effects of Chinese Herbal Medicine

**DOI:** 10.3390/jcm5060056

**Published:** 2016-06-06

**Authors:** Sai-Wang Seto, Dennis Chang, Anita Jenkins, Alan Bensoussan, Hosen Kiat

**Affiliations:** 1 National Institute of Complementary Medicine, Western Sydney University, Locked Bag 1797, Penrith 2751, NSW, Australia; s.seto@westernsydney.edu.au (S.-W.S.); 16292521@student.uws.edu.au (A.J.); a.bensoussan@westernsydney.edu.au (A.B.); 2 Faculty of Medicine, University of New South Wales, Sydney 2052, NSW, Australia; hosen.kiat@chi.org.au; 3 School of Medicine, Western Sydney University, Locked Bag 1797, Penrith 2751, NSW, Australia; 4 Faculty of Medicine and Health Sciences, Macquarie University, Sydney 2109, NSW, Australia

**Keywords:** stroke, Chinese Herbal Medicine, angiogenesis, cerebral ischemia

## Abstract

Stroke is one of the major causes of death and adult disability worldwide. The underlying pathophysiology of stroke is highly complicated, consisting of impairments of multiple signalling pathways, and numerous pathological processes such as acidosis, glutamate excitotoxicity, calcium overload, cerebral inflammation and reactive oxygen species (ROS) generation. The current treatment for ischemic stroke is limited to thromolytics such as recombinant tissue plasminogen activator (tPA). tPA has a very narrow therapeutic window, making it suitable to only a minority of stroke patients. Hence, there is great urgency to develop new therapies that can protect brain tissue from ischemic damage. Recent studies have shown that new vessel formation after stroke not only replenishes blood flow to the ischemic area of the brain, but also promotes neurogenesis and improves neurological functions in both animal models and patients. Therefore, drugs that can promote angiogenesis after ischemic stroke can provide therapeutic benefits in stroke management. In this regard, Chinese herbal medicine (CHM) has a long history in treating stroke and the associated diseases. A number of studies have demonstrated the pro-angiogenic effects of various Chinese herbs and herbal formulations in both *in vitro* and *in vivo* settings. In this article, we present a comprehensive review of the current knowledge on angiogenesis in the context of ischemic stroke and discuss the potential use of CHM in stroke management through modulation of angiogenesis.

## 1. Introduction

Stroke is a devastating disease caused by a sudden halt of blood supply to the brain due to ischemia or haemorrhage and can lead to permanent brain damage. It is one of the major causes of death and disability worldwide. Clinical and epidemiological studies have identified numerous modifiable (e.g., smoking, diabetes, hypertension and atherosclerosis) and unmodifiable (e.g., age, sex, ethical background and family history) risk factors for stroke [1]. It is widely accepted that stroke is associated with impairments of numerous signalling pathways and pathological processes, including acidosis, glutamate excitotoxicity, calcium overload, cerebral inflammation and reactive oxygen species (ROS) generation [2]. The current therapeutic approach for ischemic stroke treatment focuses on neuroprotection, thrombolysis and surgical clot removal. Despite promising results from the studies of numerous interventions in animal models of stroke, translation of these findings to the bedside has been disappointing [3]. So far, recombinant tissue plasminogen activator (tPA) is the only approved thrombolytic agent available for acute ischemic stroke [4]. However, tPA has a very narrow therapeutic window, which can only be given up to 6 h after onset of stroke and therefore only reaches less than 10% of stroke patients [5]. 

Restoring neurovascular function through re-establishing cerebral blood flow in the ischemic cerebral microvascular bed has drawn attention recently as a potential treatment strategy for ischemic stroke. During acute ischemic stroke, the reduction of tissue oxygen tension in the ischemic area often leads to a compensatory neovascularization to meet the metabolic demand. Numerous studies have shown that angiogenesis is positively correlated to the survival rate of stroke patients [6,7] indicating that modulation of the vascular growth in the ischemic area could be an important therapeutic target for ischemic stroke. Indeed, benefits of therapeutic angiogenesis (a clinical term referring to the enhancement of vessels growth in ischemic tissue) induced by direct injections or gene transfer of angiogenic factors have been demonstrated in myocardial infarction and limb ischemic injury [8]. However, similar outcomes have not been achieved in ischemic stroke. This is probably due to the unique blood supply of the brain that involves the blood–brain barrier, and the older ages of stroke patients that decrease their abilities for vascular regeneration [9]. Additionally, adverse effects associated with these angiogenic factors (e.g., hypotension and off-target tumorigenesis) are of a safety concern [8,10].

Chinese herbal medicine (CHM) has a long history of clinical use in stroke treatment. CHM often uses a variety of herbs in different complex combinations in order to strengthen the therapeutic effects (via multi-target or synergistic effects) and/or to improve their toxicity profiles. Recent clinical and large population-based epidemiological studies have demonstrated potential benefits of CHM for ischemic stroke [11,12]. Moreover, data from numerous *in vitro* and *in vivo* studies have indicated that many Chinese herbs or herbal formulations can promote angiogenesis via multiple cellular mechanisms. In this article, we present a comprehensive review of the current knowledge on angiogenesis in the context of stroke and discuss the potential use of CHM in stroke management through modulation of angiogenesis. 

## 2. Angiogenesis and Stroke

Angiogenesis refers to the sprouting of new microvessels from existing vasculature. It is an essential physiological process often stimulated by hypoxia and is required for wound healing and embryogenesis [13]. Angiogenesis relies on the coordination of a complex mix of angiogenic growth factors and inhibitors to induce and sustain endothelial cell migration and proliferation required for tissue repair over a limited time period. In humans, angiogenesis takes place at 3–4 days following ischemic insults [6]. Post-mortem analyses of brain tissue from stroke patients reveal that there was an increased cerebral blood vessel density in the penumbral areas when compared with the contralateral normal area [14]. Moreover, patients with greater cerebral blood vessel density appear to have better survival and recovery than those with lower blood vessel density [6,14]. Similarly, an increase in cerebral vessel density surrounding the infracted brain area was also observed in animal/rodent stroke models three days after the ischemic insult [15,16]. 

In stroke, following the breakdown of the blood–brain barrier, reactive oxygen species initiate morphological changes in astrocytes, forming reactive astrocytes that modify the extracellular matrix (ECM) [17] leading to complete restructure of the ECM with formation of ECM tracts [13]. These tracts are then used by migrating endothelial cells to establish new capillary buds [18]. This process is tightly controlled by a balance between angiogenic (pro-angiogenic) and angiostatic (anti-angiogenic) factors. 

A large number of angiogenic factors have been identified. These include vascular endothelial growth factor (VEGF), angiopoietins, platelet-derived growth factor (PDGF), angiogenin, transforming growth factors (TGF), basic fibroblast growth factor (bFGF), matrix metalloproteinase (MMP), nitric oxide (NO) and prostaglandin [19,20,21,22,23,24]. These factors work tightly together during the angiogenic process. Upon onset of ischemia, the combined effects of NO and VEGF cause vasodilation and increased vascular permeability leading to extravasation of plasma proteins laying down a provisional scaffold for the migration of endothelial cells for vascular sprouting [25]. This is followed by the dissociation of smooth muscle cells and loosening of the ECM. These processes are mediated by angiopoietin-2 (Ang2), an inhibitor of Tie 2 signalling, and MMP [26]. Once the sprouting path is established, proliferation and migration of endothelial cells occur which is regulated via VEGF signalling and new blood vessels are stabilised by angiopoietin-1 (Ang1) via activation of the Tie 2 receptor [27]. 

Numerous cytokines and growth factors such as interleukin-1α (IL-1α), IL-6 and thromobospondin-1 (TSP-1) have also been shown to contribute to the process of angiongesis after stroke. A decreased IL-6 level has been found in post-mortem brain tissue from post-stroke dementia patients [28]. In line with that, Gertz *et al.* (2012) showed increased cerebral lesion volumes and reduced vessel densities after an ischemic insult in IL-6(−/−) mice and that IL-6 is produced by resident brain cells to promote post-stroke angiogenesis in an *in vitro* experiment [29]. bFGF was shown to be upregulated markedly an hour after cerebral ischemia, which lasted for up to 14 days [22]. bFGF has been suggested to promote angiogenesis directly via mediating endothelial cell migration and proliferation and indirectly via upregulating VEGF in vascular smooth muscle cells [30,31].

It is important to point out that an understanding of the dynamic changes of these angiogenic factors after stroke is essential in the development of effective treatment strategies. For example, VEGF is upregulated within hours of a stroke and has a strong influence on new blood vessels growth in the injured area of the brain [32]. It has been shown that VEGF, which was given 5 min after reoxygenation following hypoxic ischemia reduced brain injury in rats [26]. Increased MMP-9 level in the early phase of stroke has been associated with increased blood brain barrier (BBB) permeability and edema [33,34]. A recent study, however, has reported a secondary phase of MMP-9 increase which was suggested to be closely associated with angiogenesis development. Inhibition of this delayed MMP-9 upregulation has been shown to cause malformed blood vessels, enlarged infarct volumes and worsened neurological deficits in mice [24]. Similarly, TSP-1 also has a biphasic upregulation after stroke with the first phase peaked at one hour and the second peak at 72 h post stroke. It has been suggested that the second peak of TSP-1 plays a vital role in cessation of angiogenesis [35]. 

Among these angiogenic factors, VEGF, an ubiquitously expressed angiogenic cytokine, has been considered to be the most important factor in regulating angiogenic processes. The VEGF gene family comprises seven members including placental growth factors, VEGF A, VEGF B, VEGF C, VEGF D, VEGF E and VEGF F, each member containing a signal sequence that cleaves during its biosynthesis. There are at least four possible splice/isoform variants of VEGF that exist *in vivo*, which include VEGF165, VEGF 206, VEGF 189 and VEGF 121 [36]. VEGF binds to its receptors on vascular endothelial cells to directly initiate an angiogenic response. The VEGF family ligands have three receptor protein kinases which include VEGFR-1 (fms-like tyrosine receptor, Flt-1), VEGFR-2 (fetal liver kinase/kinase insert domain receptor, Flk-1) and VEGFR-3 [37]. Both VEGFR-1 and -2 are expressed on vascular endothelial cells while VEGFR-3 is mostly expressed on lymphatic endothelial cells [38]. It has been suggested that VEGF is upregulated during ischemia through a post-transcriptional mechanism [39]. It has been shown that transcription factors signal transducers and activators of transcription (STAT)-1 and STAT-3 regulate VEGF expression by modulation of the hypoxia induced factor (HIF)-1 in vascular smooth muscle cells [40]. Interestingly, although VEGF has a stronger binding affinity to VEGFR-1 than VEGFR-2, most of the biological effects of VEGF are mediated by VEGFR-2 [41]. Activation of the VEGFR-1 and -2 promotes endothelial cell proliferation and migration, and stimulates ECM degradation, which increases vascular permeability [42,43,44,45]. These VEGF-induced processes are mediated by multiple downstream signalling pathways, such as PI3K/Akt and MEK/ERK protein kinase pathways [19,46]. Moreover, NO has been shown to upregulate VEGF expression in endothelial cells, contributing to the VEGF-mediated vascular permeability increase and vasodilatory in ischemic brain [47]. Studies have shown that this increased vascular permeability predominantly occurs in the first hours of ischemia [43,47]. As such, it has been suggested that the role of acute angiogenesis is for the infiltration of macrophages and phagocytosis of necrotic debris (clean-up hypothesis) [48]. 

Recently, the role of miRNAs in mediating angiogenesis has been studied extensively [49]. Interestingly, overexpression of miR-210 has been found to be associated with angiogenesis in the brain of adult non ischemic mice with an up-regulation of VEGF [50]. In a rat model of middle cerebral artery occlusion, miR-139 and miR-335 were found to be down-regulated in the endothelial cells [51,52]. Both miR-139 and miR-335 are closely associated with stroke-induced angiogenesis via ROCK1 and ROCK2 signalling (both ROCK1 and ROCK2 are VEGF signalling regulators) [49]. miR-15a has been found to be upregulated in cerebral vessels suppressing stroke-induced angiogenesis via inhibition of endothelial fibroblast growth factor 2 (FGF2) and VEGF in a mouse model of focal cerebral ischemia [35]. It has also been shown that miR-150 and miR-140-5p play an important role in regulating VEGF signalling during angiogenesis. miR-140-5p has been suggested to modulate angiogenesis via upregulation of VEGFA expression in hypoxic endothelial cells, an effect by directly targeting the 3′ untranslated region of VEGFA [53]. Upregulation of miR-150 has been found to reduce vascular density in the infarct area in a rat stroke model. Furthermore, *in vitro* work from the same study showed that miR-150 decreased endothelial cells proliferation and migration via downregulation of VEGF expression [54]. 

In addition to VEGF, other angiogenic factors such as angiopoietin (Ang) and thrombospondins (TSPs) have been shown to be up-regulated during ischemia [21,55]. Ang mediates stabilization and remodelling of new-born blood vessel by binding to Tie-2 receptors while TSPs acts on its receptor, CD36, to control mitosis, migration and apoptosis [56,57].

Although there is ample evidence demonstrating a correlation between angiogenesis and survival of stroke patients, the post-ischemic angiogenesis is short lived. The restorative function of angiogenesis appears to take effect 48 h after the initial ischemic insult and often only lasts for a few weeks [43]. This could explain why functional improvement usually occurs during the early months after the ischemic incidence in most stroke survivors [58]. The effects of enhancing this restorative angiogenesis on ischemic outcome remain to be explored. 

## 3. Angiogenic Effects of Chinese Herbal Medicine in Ischemic Stroke

### 3.1. Models of Angiogenesis Used in CHM Research

A variety of pre-clinical models have been used in the evaluation of the cellular and molecular signalling mechanisms underlying angiogenesis and effectiveness of pro-angiogenic or anti-angiogenic treatments. Some of the most commonly used methods for the evaluation of angiogenic effects of CHMs are discussed below. 

### 3.2. In Vitro Quantitation of Angiogenesis

Angiogenesis involves the stimulation of endothelial cell migration and proliferation. Therefore, current *in vitro* models of angiogenesis are mostly performed in cultured vascular endothelial cells [59]. The common endothelial cell-based models used in CHM research include migration, invasion, cell proliferation and tube formation assays. For example, Zhang *et al.* evaluated the angiogenic effects and the underlying mechanisms of *Radix astragali*, a commonly used Chinese herb for the treatment of cardiovascular diseases using a combination of proliferation, migration, invasion and tube formation assays in human umbilical vein endothelial cells (HUVECs). This study demonstrated that *Radix astragali* extract promoted cell proliferation, migration, and capillary formation in HUVECs potentially through enhancing the mRNA expression of VEGF [60]. 

The main weakness of these *in vitro* cell-based models is that they can only be used to evaluate some aspects of the very complicated angiogenic process. However, because of their rapid and relative inexpensive nature, these assays are invaluable tools to understand the underlying cellular and molecular mechanisms of angiogenesis and different levels of the angiogenic cascade. The use of human cell lines such as human cerebral microvascular endothelial cell has also made these models directly relevant to the study of angiogenesis of CHMs for human diseases. 

### 3.3. In Vivo Quantitation of Angiogenesis

*In vivo* angiogenic methods are crucial for assessing the overall outcome of angiogenesis and are also the only possible way to study toxicity, immune responses and biotransformation of pro-angiogenic or anti-angiogenic agents [59]. Transgenic zebrafish expressing green fluorescent protein (GFP) labelled vascular endothelial cells is one of the most important *in vivo* animal models to study angiogenesis. A large number of transgenic lines such as Tg(kdr:RFP)la4 and Tg(kdr:G-RCFP) [61] have been developed. High-resolution long-term time-lapse analysis of cell division and positioning of vessel development have been achieved using enhanced GFP (e.g., Tg(flk1:eGFP) and Tg(fli:eGFP)) and nucleus localised GFP (e.g., Tg(fli1:neGFP)) in zebrafish [62]. Owing to its small size, cheap maintenance and rapid embryo reproduction, zebrafish have been used as a preferred high-throughput model of angiogenesis in recent years for drug screening and the study of herbal and natural compounds [63]. For example, the angiogenic effect of saponin extract of *Panax notoginseng* has been shown to promote the growth of subintestinal vessels in zebrafish [64].

Advancements in imaging have massively transformed our way to evaluate angiogenic effects of drugs in animal models. Imaging systems such as ultrasonography are used to monitor angiogenesis continuously in the same animal over time. For example, microbubble contrast agents are injected into animal for visualisation of blood vessels using ultrasonography. Angiogenesis is monitored with a morphometric analysis program, which measures the amount of microbubbles at the regions of interest [65]. Antibody labelled microbubbles (e.g., VEGFR2) have also been developed to provide high resolution vessel specific images [66]. In addition, confocal and multiphoton microscopy, magnetic resonance imaging (MRI), computed tomography (CT) and positron emission tomography (PET) have been applied to assess angiogenesis in both animals and humans [67]. VEGFR/VEGFR-2 mediated cerebral neurovascular regeneration has been demonstrated in an ischemic stroke model of mice by measuring local cerebral blood flow using laser scanning imaging technique [68]. Similarly, using video microscopy and a laser Doppler perfusion imaging method, Wu *et al.* (2014) showed that *Pueraria lobata* could prevent stroke by increasing the blood flow perfusion in the pia mater of the spontaneously hypertensive rats [69]. These methods offer a minimal invasive procedure with high reproducibility. Another major advantage of the vascular imaging models is their ability to measure blood flow and vascular permeability, and to analyse cellular and molecular changes in the blood vessel walls, allowing elucidation of any structural and functional abnormalities of the newly formed blood vessels. However, expensive and sophisticated equipment and specialised skills personnel are required in these models.

### 3.4. Chinese Herbal Medicine Used in Ischemic Stroke

CHM has a long history of clinical use for stroke prevention, treatment and rehabilitation [70]. Data from numerous stroke clinical studies have demonstrated the potential benefits of Chinese herbs with a high level of flavonoids and sulphur compounds [71,72]. It has been suggested that CHM improve neurological function after stroke primarily via their anti-thrombogenic, anti-oxidative and anti-inflammatory properties. However, recent studies have highlighted the important role of CHM in facilitating angiogenesis during the neurovascular regeneration in response to stroke. For example, the beneficial effects of green tea in preventing ischemic stroke [11] could be partly explained by the angiogenic effect of eipgallocatechin-3-gallate (EGCG), the most abundant catechin in green tea. A recent *in vitro* study has demonstrated that EGCG promoted endothelial cell proliferation, migration and tube formation through activation of the transient receptor potential vanilliod type (TRPV1) calcium channel [73]. Some common Chinese herbs and herbal formulations that possess angiogenic effects are summarised in Table 1. 

It is worth noting that some Chinese herbs can elicit diverse pharmacological actions on angiogenesis. For example, EGb 761 (a standardised extract of *Ginkgo biloba*) has been shown to upregulate VEGF mRNA expression and activity, promoting angiogenesis in a permanent ischemic stroke model in mice [74], while an *in vitro* study has shown possible anti-angiogenic effects of *Ginkgo biloba* through down-regulation of VEGF mRNA expression in THP-1 cells [75,76] indicating potential different mechanisms of action of EGb 761 in different models. Resveratrol, a polyphenol found in abundance in the skin of grapes, blueberries and raspberries, has also demonstrated multiple actions on angiogenesis. It has been shown that resveratrol enhanced angiogenesis in both small animal and cultured cell models [77,78]. However, a more recent study demonstrated that a high dose of resveratrol (100 mg/kg/day orally) failed to increase collateral density in ischemic myocardium in a swine model of metabolic syndrome [79]. This discrepancy could be, at least partially, due to an enhanced angiostatin and thrombospondin-1 production in response to the higher dose of resveratrol [79]. Some of the bioactive components of *Panax ginseng* (e.g., ginsenosides including Rb1 and Rg1), have also demonstrated opposing effects on angiogenesis. Rg1 was found to stimulate angiogenesis via induction of VEGFR-2 and VEGF expression while Rb1 was shown to inhibit endothelial cell migration in *in vitro* studies [80,81,82]. Moreover, an anti-angiogenic effect was demonstrated when Rg1 and Rb1 were combined in equal concentrations or when Rb1 was greater than Rg1 [82,83].

### 3.5. Mechanisms of Action Underlying the Angiogenic Effects of CHM

In recent years, there is an increasing interest in the pro-angiogenic effects of CHM for managing stroke. A large number of *in vitro* and *in vivo* pre-clinical studies have been conducted to assess the mechanisms of action underlying the pro-angiogenic effects of CHM in ischemic stroke. Multiple signal transduction pathways have been identified to be associated with the CHM-mediated angiogenesis. Given the important role of VEGF in regulating angiogenesis, it is not surprising that many Chinese herbs elicit their pro-angiogenic activities via the VEGF signalling pathways. *Astragalus membranaceus* extract has been shown to stimulate endothelial cell proliferation and tube formation. The key bioactive component of the herb, astragaloside IV (AS-IV), was suggested to be responsible for the effects via both VEGF- and Akt-dependent signalling pathways [60,84]. In a VEGFR tyrosine kinase inhibitor II (VRI)-induced blood vessel damage zebrafish model, polysaccharides from *Astragalus membranaceus* reduced blood vessel lose by reversing the VRI-induced down-regulation of Flk-1 and Flt-1 mRNA expression, confirming their modulatory effects on the VEGF-VEGFR2 signalling pathway [85]. Similarly, cornel iridoid glycoside, a bioactive component of *Cornus officinalis*, has been shown to promote angiogenesis and neurogenesis via up-regulation of VEGF and Flk-1 mRNA and protein expression in a focal cerebral ischemia rat model [86].

Chinese herbs have been shown to promote angiogenesis via other signalling mechanisms. Calycosin, a major isoflavonoid isolated from *Astragalus membranaceus*, promotes angiogenesis through activation of both the VEGF-VEGFR2 and mitogen-activated protein kinase (MAPK)/extracellular signal-regulated kinase (ERK) signalling pathway [87]. Similarly, resveratrol has also been suggested to promote angiogenesis and neurovascular recovery after stoke via activation of the MAPK/ERK signalling [88]. In addition, resveratrol was shown to be partly associated with an elevated NO level resulting in an increase in VEGF and matrix metalloproteinase (MMP) level. NO is one of the major downstream mediators of many vascular growth factors [89]. In stroke, NO has been considered as a two-edged sword -NO generated by endothelial NOS (eNOS) exerting neuro and vascular protective effects while NO produced by neuronal NOS (nNOS) and inducible NOS (iNOS) inducing neurotoxicity effects [90,91]. Ginsenoside Rg1, an active component of ginseng, has been demonstrated to down-regulate miR-214 expression, leading to increased eNOS expression [92]. 

Hypoxia-inducible factor (HIF-1) induces angiogenesis via upregulation of VEGF in ischemic brain in response to the change of intracellular oxygen level [93]. Danshen (*Salvia miltiorrhiza*) injection has been shown to improve hypoxia-induced angiogenesis in mice via increased expression of HIF-1 and VEGFA [94]. Ginsenoside Rg1 has been suggested to facilitate brain repair by modulating angiogenesis and neurogenesis targeting HIF-1 [95,96]. Furthermore, an *in vitro* study showed that ginsenoside Rg1 regulated HIF-1 expression through PI3K/Akt and its effector p70 S6 kinase [81]. 

Stem cell therapy has recently emerged as a promising strategy for ischemic stroke [97]. A number of recent studies have demonstrated potential clinical benefits of the combination of CHM and bone marrow stromal cells (BMSCs) for the treatment of stroke [96]. For instance, it has been shown that a combined therapy of astragaloside IV and BMSC generated better neurological outcomes in a rat cerebral ischemic model. These effects were associated with improved BMSC survival and migration induced by the astragaloside IV-induced VEGF generation [96]. In addition, Tongxinluo (TXL), a complex herbal formula, has been shown to promote MSCs tube formation via upregulation of MMP-2 and VEGF expression in an *in vitro* study [98].

### 3.6. Angiogenic Effects of Complex Herbal Formulations

Several Chinese herbal formulations have been shown to protect brain damage after ischemic stroke. Tongxinluo (TXL), a complex herbal formula consisting of *Panax ginseng*, *Mesobuthus martensii*, *Hirudo nipponica*, *Eupolyphaga sinensis*, *Scolopendra subspinipes*, *Periostracum cicadae*, *Paeonia lactiflora*, *Ziziphus spinosa*, *Dalbergia odorifera*, *Santalum album and Dryobalanops aromatica* [99], is widely used for the treatment of cardio-cerebrovascular diseases [99,100]. TXL has been shown to exert protective effects against cerebral damage and neuronal apoptosis via PI3K/Akt signalling pathway in a rat model of focal cerebral ischemia and reperfusion injury [101]. Additionally, TXL has also been suggested to induce pro-angiogenic effects [102]. For example, in a study using middle cerebral artery occlusion (MCAO) model in rats, TXL significantly improved neurological function via promoting neurogenesis and angiogenesis in the ipsilateral thalamus [103]. A recent study by the same group showed that TXL promoted endothelial cells proliferation in the subventricular zone and vascularisation in the peri-infarct area in a rat stroke model [104]. It is worth noting that, in these studies, TXL was administrated 24 h post ischemic insult, highlighting its potential in post-stroke treatments. 

Buyang Huanwu Decoction (BYHWD) is a 7-herb Chinese herbal formation consisting of *Astragalus membranaceus*, *Angelica sinensis*, *Paeonia lactiflora*, *Ligusticum chuanxiong*, *Prunus persica*, *Carthamus tinctorius* and *Lumbricus terrestris*. The formulation is widely used for treating ischemic and neurodegenerative diseases [105,106]. Results from pervious clinical studies have shown that BYHWD improved neurological functions in cerebral infraction patients, which has been suggested to be associated with an increased serum VEGF expression [107]. In an animal study, BYHWD combined with mesenchymal stem cells transplantation increased cerebral vascular density in rat brain after bilateral carotid artery ligation via an augmentation of VEGF and Ki-67b expressions induced by exosomes in endothelial cells [108]. Similarly, BYHWD has been shown to promote neurological recovery and angiogenesis via VEGFR-2 activation through the PI3K/Akt signalling pathway in a mouse model of intracerebral haemorrhage [109]. 

Danggui-Shaoyao-San (DSS), a 6-herb Chinese herbal formulation consisting of *Angelica sinensi*, *Paeonia lactiflora*, *Poria cocos*, *Atractylode smacrocephala*, *Alisma orientals* and *Ligusticum chuanxiong*, is widely used in China, Korea and Japan to relieve menorrhagia and other abdominal pains of woman via estrogen modulation [110]. However DSS has been shown to reduce infract size 24 h post-stroke in a mouse model of MCAO [111] and to increase cerebral blood flow in the hippocampus in a rat model of global cerebral ischemia [112]. In a recent study, Ren *et al.* (2015) demonstrated that these post-ischemic protective effects of DSS are associated with focal angiogenesis and neurogenesis via up-regulation of VEGF protein expression and increased eNOS activity [113]. In a mouse model of ageing, DSS demonstrated a neuroprotective effect by ameliorating oxidative stress and neuronal apoptosis via the Bcl-2/Bax and caspase-3 signalling in the brain [114]. 

## 4. Conclusions and Future Directions

Substantial effort has been invested in identifying and developing novel interventions for the treatment and prevention of stroke. Recent studies have shown the therapeutic potential of pro-angiogenic agents for ischemic stroke. A number of Chinese herbs or herbal formulations have demonstrated significant neuroprotective and pro-angiogenic effects and may offer viable treatment options for the disease especially during the early stage of rehabilitation. However, the overall scientific evidence to support the use of CHMs for stroke treatment remains scarce and the results of these studies are often inconclusive and contradictory. The underlying reasons for this inconsistency are multiple, but primarily due to the complex chemical and pharmacological properties of CHMs. In addition, the interactions (synergism) between the multiple bioactive components of CHMs add another level of complexity. More research is needed to gain a better understanding of the mechanisms of action underlying these interventions, key bioactive components responsible for the effects, and interactions among these components (synergism). Current experimental models available are designed for single-entity, single-target interventions and are not suited for the study of synergistic mechanisms underlying complex herbal formulations. New methods (such as system biology) are urgently needed for the study of angiogenic properties of CHM. Additionally, the angiogenic effects of CHM may sometimes be masked by other mechanisms (e.g., neurogenesis) during tissue repair after stroke, and therefore more work using robust and sensitive models is required to differentiate these effects. Finally, despite a long history of clinical use of CHM in the treatment of stroke, clinical evidence is still generally lacking. More rigorously designed randomized, controlled trials are required to further validate CHMs efficacy. The possible side effects and potential risk of angiogenesis-borne diseases such as formation and metastasis of tumour (e.g., breast cancer) and aggravation of diabetic retinopathy should also be considered when applying this strategy for ischemic stroke.

## Figures and Tables

**Table 1:** Summary of the pro-angiogenic effects of common Chinese herbs and herbal formulations.

**Herb**	**Phytochemistry**	**Treatment Methods and Dosage**	**Models of Study**	**Mechanism of Action**	**References**
*Angelica sinensis* (Dang Gui)	Ferulic acid	0.1 μg/mL—10 μg/mL; 24 h	Proliferation, DNA synthesis and cell cycle distribution assay (ECV304)	↑VEGF; ↑Cyclin D1	[115]
*Cornus officinalis* (Sahn Zhu Yu)	Cornel iridoid glycoside	20, 60 and 180 mg/kg/day intragastrically, 3 h after onset of MCAO	MCAO in rat; angiogenesis assessed 7, 14 and 28 days after ischemia	↑VEGF and Flk-1	[86]
*Ginkgo biloba* (Yin Xing)	EGb 761	50 mg/kg/day (i.p.) for 10 days	Acellular nerve allografts in rat; angiogenesis assessed 2 and 4 weeks	↑VEGF; ↑SOX18; ↑Prom 1; ↑IL-6	[74,116]
*Panax notoginseng* (San Qi)	Ginsenoside Rg-1	5 mg/kg/day (i.p.) for 7 days	AMI in rat; microvessel density of infraction area assessed after 4 weeks	↑eNOS; ↑VEGF; ↑MET tyrosine kinase receptor; ↑Hypoxia-inducible factor (HIF-1)	[92,117,118]
	Saponin	0.03–100 μg/mL	Human umbilical vein endothelial cells (HUVECs) proliferation, migration and tube formation assay and zebrafish (assessed 72 hpf)	↑VEGF-KDR/Flk-1 and ↑PI3K-Akt-eNOS signaling pathways	[64]
	Notoginsenoside	0.25 mg/kg, 2.5 mg/kg and 25 mg/kg (i.p.) for 28 days	HUVECs proliferation, migration and tube formation assay and wound healing assay in mice	PI3K/AKT and MAPK/ERK signalling	[119]
*Astragalus membranaceus* (Huang Qi)	Extract	0.8–100 μg/mL	Human umbilical vein endothelial cells (HUVECs) proliferation, migration and tube formation assay and zebrafish (assessed 72 hpf)	↑VEGF signalling	[60]
	Astragalosdie IV (AS-IV)	0.1–100 μg/mL	Human umbilical vein endothelial cells (HUVECs) proliferation, migration and tube formation assay and zebrafish (assessed 72 hpf)	↑VEGF; Akt-dependent signalling; JAK2/STAT3 signalling; ERK1/2 signalling	[84,120]
	Polysaccharides	10–300 μg/mL	VRI-induced blood vessel loss in zebrafish (21 and 45 h after induction)	↑Flk-1 and Flt-1; ↑VEGF-VEGFR2 signalling	[85]
	Calycosin	10–100 μM	Human umbilical vein endothelial cells (HUVECs) proliferation, migration and tube formation assay and zebrafish (assessed 72 hpf)	selective estrogen receptor ; ↑VEGF-VEGFR2 signalling; ↑MAPK/ERK signalling	[87]
*Rehmannia glutinosa* (Di Huang)	Extract	2–62.5 μg/mL	Human microvascular endothelial cells (HMEC-1) proliferation, migration and tube formation assay and zebrafish (assessed 72 hpf)	↑VEGF, ↑VEGFR-3, MMP-9 and ANGPT-1	[121]
*Salvia miltiorrhiza* (Dan Shen)	Extract	3 g/kg/day or 6 g/kg/day (i.p.) for 28 days	Mouse model of myocardial infarction-induced cardiac damage; Angiogenesis assessed 4 weeks after induction	↑VEGFA; ↑HIF-1	[94]
	Salvianolic acid A	0.3, 30 and 30 mg/L (6–72 h)	Endothelial progenitor cells proliferation and migration assay and chick embryo chorioallantoic membrane model	↑VEGF, VEGFR-2, and MMP-9	[122]
	Tanshinones (Compounds 1–10)	0.003–1 μM	VRI-induced blood vessel loss in zebrafish (assessed at 48 or 72 hpf)	↑PI3K signalling; ↑MAPK signalling	[123]
Skin of grapes, blueberries and raspberries	Resveratrol	0.1–10 μM	Cerebral endothelial cells with resveratrol promoted proliferation, migration and tube formation assays	↑VEGF; ↑MAPK/ERK signalling; ↑NO; ↑MMP	[88,89]
**Formulation**	**Compositions**	**Treatment methods and Dosage**	**Models of Study**	**Mechanism of action**	**References**
Buyang huanwu decoction	*Astragalus membranaceus*, *Angelica sinensis*, *Paeonia lactiflora*, *Ligusticum chuanxiong*, *Prunus persica*, *Carthamus tinctorius*, *Lumbricus*	4.36 g/kg/day intragastrically (24 h after ICH) for 7 days	Intracerebral hemorrhage (ICH) model of mouse (Assessed at day 1, 3 and 7 after ICH)	↑VEGFR2; ↑PI3K/Akt signaling; ↑angiopoietin-1 (ANGPT-1)	[109,124]
Danggui-Shaoyao-San	*Paeonia lactiflora*, *Angelica sinensis*, *Ligusticum chuanxiong*, *Poria cocos*, *Atractylodis macrocephalae* and *Alisma orientalis*	600 mg/kg/day intragastrically for 14 days	MCAO in rat; angiogenesis assessed 14 days after ischemia	↑eNOS; ↓oxdiative stress; ↓Bcl-2/Bax; ↓Caspase-3	[113,114]
Qiliqiangxin	*Astragalus membranaceus*, *Aconitum carmichaeli*, *Panax ginseng*, *Salvia miltiorrhiza*, *Lepidium apetalum*, *Periploca sepium*, *Alisma orientalis*, *Carthamus tinctorius*, *Polygonatum odoratum*, Seasoned Orange Peel, and *Cinnamomum cassia*	0.25, 0.5 and 1.0 g/kg/day intragastrically for 6 weeks	Rat model of myocardial infarction; assessed 6 weeks after induction	↓Bcl-2/Bax; ↑Akt-dependent signalling pathways; ↑HIF-1	[125]
Tongxinluo	*Panax ginseng*, *Mesobuthus martensii*, *Hirudo*, *Eupolyphaga seu steleophaga*, *Scolopendra subspinipes*, *Periostracum cicadae*, *Paeonia lactiflora*, *Ziziphus spinosa*, *Dalbergia odorifera*, *Santalum album*, and *Dryobalanops aromatica*	0.4 g, 0.8 and 1.6 g/kg/day orally three times a day for three days before MCAO and until endpoint	MCAO in rat; angiogenesis assessed 1, 3, 5 and 7 days after ischemia/reperfusion	↑VEGF; ↑PI3K/Akt signalling	[101,102]
Xiongshao Capsule	*Ligusticum chuanxiong* and *Paeonia lactiflora*	Serum from collected from rat received 41.7, 83.3 mg/kg and 166.6 mg/kg twice a day for 7 days	Human umbilical vein endothelial cells (HUVECs) proliferation, migration and tube formation assays	↑VEGF; ↑basic fibroblast growth factor (bFGF)	[126]
Xuefu Zhuyu Decoction	*Angelica sinensis*, *Bupleurum chinensis*, *Carthamus tinctorius*, *Citrus aurantium*, *Cyathula officinalis*, *Glycyrrhiza glabra*, *Ligusticum chuanxiong*, *Paeonia lactiflora* Pall., *Platycodon grandiflorus*, *Prunus persica*, *Rehmannia glutinosa Liboschitz*	XZD-containing human serum	Human microvascular endothelial cells (HMEC-1) proliferation, migration, adhesion and tube formation assay (assessed at 24, 28 and 72 h)	↑VEGF signalling; ↑NO	[127]
Xuesetong Soft Capsules	*Panax notoginseng* total saponin extract	0.4 g/kg/day intragastrically for 6 weeks	Rat model of myocardial infarction; assessed 6 weeks after induction	↑VEGF	[128]
